# Supplementary material for: Hybrid Models and Biological Model Reduction with PyDSTool
Source: PLoS Comput Biol. 2012 Aug 9;8(8):e1002628. doi: 10.1371/journal.pcbi.1002628 (PMC3415397; doi:10.1371/journal.pcbi.1002628)
Supplement: Text S4 — Complete source code for the PyDSTool package (version 0.88.120504). Includes API documentation and help files linking to web pages. This file is identical to the current public release on Sourceforge.net. (ZIP) [file pcbi.1002628.s004.zip › PyDSTool/html/PyDSTool.ModelConstructor'-pysrc.html]

xml version="1.0" encoding="ascii"?


PyDSTool.ModelConstructor'


| Home | Trees | Indices | Help | PyDSTool |
| --- | --- | --- | --- | --- |

Package PyDSTool ::
Module ModelConstructor'

[hide private]

[frames] | no frames]

# Source Code for Module PyDSTool.ModelConstructor'

```
   1  """Model Constructor classes.
 
   2  
 
   3     Instantiate abstract Model Specifications into concrete simulation code.
 
   4  
 
   5     Robert Clewley, September 2005.
 
   6  
 
   7  
 
   8  Overview of steps that ModelConstructor takes:
 
   9  
 
  10  1. Build Generators for each vector field
 
  11   * Take flattened spec and select a compatible Generator type and language
 
  12     * include check that compatibleGen selection actually exists,
 
  13        and is compatible with specType/domain, and with cts/discrete
 
  14        time variables)
 
  15     * presently, each v.f. must have all the same Gen type
 
  16   * Map things like x^y and x**y power specs -> pow(x,y)
 
  17      and abs() function -> fabs() in C.
 
  18  
 
  19  2. Given the specific target lang/Generator with a ModelSpec vector field
 
  20   * Associate additional events, code inserts, reused vars
 
  21   * Choose which domains map to domain verification events (certainly none that
 
  22      are infinite -- although semi-infinite can be checked in one direction)
 
  23   * All non-infinite domain ends would have an event created for them, but
 
  24      default is for them to be inactive.
 
  25  
 
  26   The 'mspec' argument to the GeneratorConstructor class must be a complete
 
  27   ModelSpec (up to introduction of global references including definitions of
 
  28   external inputs).
 
  29  
 
  30   The resulting model output from getModel contains the ModelSpec mspec in
 
  31   order to use its structure in resolving information about the relationship
 
  32   between variables.
 
  33  """ 
  34  
 
  35  # PyDSTool imports
 
  36  from errors import * 
  37  from common import * 
  38  from utils import info, remain, intersect 
  39  import Model, Generator, ModelSpec, Symbolic, Events, MProject 
  40  from parseUtils import symbolMapClass, NAMESEP, isNumericToken 
  41  
 
  42  # Other imports
 
  43  from numpy import Inf, NaN, isfinite,  array, \
 
  44       arange, zeros, ones, concatenate, swapaxes, take, \
 
  45       sometrue, alltrue, any, all 
  46  import numpy, scipy, math # for access by user-defined code of EvMapping 
  47  import sys, types, copy 
  48  
 
  49  # Exports
 
  50  __all__ = ['GeneratorConstructor', 'ModelConstructor', 'makeModelInfo',
 
  51             'makeModelInfoEntry', 'embed', 'EvMapping', 'makeEvMapping',
 
  52             'GDescriptor', 'MDescriptor'] 
  53  
 
  54  # -----------------------------------------------------------------------------
 
  55  
 
  56  mathNameMap = dict(zip(Symbolic.allmathnames_symbolic,
 
  57                         Symbolic.allmathnames)) 
  58  
 
  59  
 


60 -class Descriptor(args):


61      """Abstract class for model and generator descriptors""" 
  62      _validKeys = () 
  63      _defaults = {}  # for values other than None 
  64      _checkKeys = ()  # values that must be set in order for the generator to be instantiable 
  65  
 


66 -    def __init__(self, **kw):


67          self.__dict__ = filteredDict(kw, self._validKeys) 
  68          if remain(kw.keys(), self._validKeys) != []: 
  69              print "Valid keys: ", self._validKeys 
  70              raise ValueError("Invalid keys provided for Model Descriptor") 
  71          done_defs = [] 
  72          for def_key in remain(self._defaults.keys(), kw.keys()): 
  73              def_value = self._defaults[def_key] 
  74              done_defs.append(def_key) 
  75              self.__dict__[def_key] = def_value 
  76          for key in remain(self._validKeys, kw.keys()+done_defs): 
  77              self.__dict__[key] = None

  78  
 


79 -    def validate(self):


80          raise NotImplementedError("Defined in concrete sub-class")

  81  
 


82 -    def __repr__(self):


83          return className(self)

  84  
 
  85      __str__ = __repr__

  86  
 
  87  
 
  88  
 


89 -class GDescriptor(Descriptor):


90      """All-in-one descriptor class for single Generators, and information
 
  91      necessary to be able to build a Model object using a ModelConstructor call
 
  92      -- i.e. for forming a valid 'generatorspecs' field.
 
  93      """ 
  94      _validKeys = ('changelog', 'orig_name', 'modelspec', 'description',
 
  95                    'algparams', 'target', 'withStdEvts', 'stdEvtArgs', 'withJac',
 
  96                    'withJacP', 'reuseTerms', 'eventPars', 'unravelInfo',
 
  97                    'userEvents', 'userFunctions', 'userEventMaps', 'options') 
  98      _defaults = {'description': '', 'withStdEvts': False, 'withJac': False,
 
  99                   'withJacP': False, 'unravelInfo': True, 'options': {}} 
 100      _checkKeys = ('target') 
 101  
 
 102  #    def __init__(self, **kw):
 
 103  #        Descriptor.__init__(self, **kw)
 
 104  #        self.component_hierarchy = GTree(self.modelspec)
 
 105  
 


106 -    def __getitem__(self, hier_name):


107          """Return object in model spec named using the hierarchical
 
 108          naming format.
 
 109          """ 
 110          return self.modelspec[hier_name]

 111  
 


112 -    def search(self, hier_name):


113          return self.modelspec.search(hier_name)

 114  
 


115 -    def validate(self):


116          validated = isinstance(self.modelspec, ModelSpec.ModelSpec) and \
 
 117                    self.modelspec.isComplete() and \
 
 118                    self.modelspec.isDefined(ignoreInputs=True) 
 119          freeSymbols = self.modelspec.freeSymbols 
 120          return (validated, freeSymbols)

 121  
 


122 -    def isinstantiable(self):


123          return self.validate()[0] and self.target is not None

 124  
 
 125  
 
 126  
 


127 -class MDescriptor(Descriptor):


128      """All-in-one descriptor class for hybrid model definitions and information
 
 129      necessary to be able to build a Model object using a ModelConstructor call.
 
 130  
 
 131      generatorspecs should be a dictionary of gen modelspec names -> modelspecs.
 
 132      """ 
 133      _validKeys = ('changelog', 'orig_name', 'name', 'generatorspecs',
 
 134                    'description', 'abseps', 'activateAllBounds',
 
 135                    'checklevel', 'tdata', 'indepvar', 'icvalues', 'parvalues',
 
 136                    'inputs', 'unravelInfo',
 
 137                    'userevents', 'userfns', 'reuseTerms',
 
 138                    'withJac', 'withJacP',
 
 139                    'eventtol', 'eventPars',
 
 140                    'withStdEvts', 'stdEvtArgs') 
 141      _defaults = {'description': '', 'indepvar': ('t', [-Inf,Inf]),
 
 142                   'checklevel': 2, 'activateAllBounds': False,
 
 143                   'generatorspecs': {}, 'icvalues': {}, 'parvalues': {},
 
 144                   'inputs': {}, 'unravelInfo': True} 
 145      _checkKeys = ('icvalues', 'parvalues', 'inputs') 
 146  
 


147 -    def validate(self):


148          assert hasattr(self.generatorspecs, 'values') and \
 
 149                 hasattr(self.generatorspecs, 'keys') 
 150          validated = alltrue([isinstance(gd, GDescriptor) for \
 
 151                               gd in self.generatorspecs.values()]) 
 152          # !!!TO DO!!!
 
 153          # Check for consistency of any internal interfaces defined
 
 154          inconsistencies = [] 
 155          return (validated, inconsistencies)

 156  
 


157 -    def isinstantiable(self, verbose=False):


158          valid = self.validate()[0] 
 159          vars_i_all = True 
 160          pars_i_all = True 
 161          inps_i_all = True 
 162          for ms in self.generatorspecs.values(): 
 163              all_vars = ms.modelspec.search('Var') 
 164              dyn_vars = [v for v in all_vars if ms.modelspec._registry[v].obj.specType == 'RHSfuncSpec'] 
 165              vars_i = alltrue([varname in self.icvalues for \
 
 166                                varname in dyn_vars]) 
 167              pars_i = alltrue([(parname in self.parvalues or \
 
 168                                 par.spec.specStr !='') \
 
 169                            for parname, par in ms.modelspec.pars.items()]) 
 170              inps_i = alltrue([inpname in self.inputs for \
 
 171                                inpname in ms.modelspec.inputs]) 
 172              if verbose: 
 173                  if not vars_i: 
 174                      print ms.modelspec.name, "Some ICs missing" 
 175                  if not pars_i: 
 176                      print ms.modelspec.name, "Some param values missing" 
 177                  if not inps_i: 
 178                      print ms.modelspec.name, "Some input values missing" 
 179              vars_i_all = vars_i_all and vars_i 
 180              pars_i_all = pars_i_all and pars_i 
 181              inps_i_all = inps_i_all and inps_i 
 182          return valid and vars_i_all and pars_i_all and inps_i_all

 183  
 


184 -    def get_desc(self, name):


185          if name in self.generatorspecs: 
 186              return self.generatorspecs[name] 
 187          else: 
 188              raise KeyError('Generator %s does not exist in registry'%name)

 189  
 


190 -    def add(self, gd):


191          self.generatorspecs[gd.modelspec.name] = gd

 192  
 
 193  
 
 194  # ------------------------------------------------
 
 195  
 
 196  
 


197 -class GeneratorConstructor(object):


198 -    def __init__(self, mspec=None, userevents=None, userfns=None,
 
 199                   unravelInfo=True, inputs=None, checklevel=2,
 
 200                   activateAllBounds=False, activatedBounds=None,
 
 201                   targetGen="", algparams=None, indepvar=('t',[-Inf,Inf]),
 
 202                   tdata=None, parvalues=None, icvalues=None, reuseTerms=None,
 
 203                   options=None, abseps=None, eventPars=None, preReuse=False,
 
 204                   preReuseTerms=None, preFlat=False):


205          """Notes for initialization arguments:
 
 206  
 
 207          mspec : corresponding ModelSpec, for reference
 
 208  
 
 209          userevents : list of Event objects
 
 210          userfns : dictionary of named user functions specs
 
 211          inputs : dictionary of Variable objects
 
 212          algparams : dictionary of algorithmic parameters for Generator
 
 213          parvalues : dictionary of parameter values
 
 214          icvalues : dictionary of initial condition values
 
 215          reuseterms : dictionary of reused terms in specifications
 
 216  
 
 217          targetGen : STRING name of any compatible Generator class, e.g. 'Vode_ODEsystem'
 
 218  
 
 219          eventPars : list of parameter names associated solely with events
 
 220  
 
 221          options : Internal use by ModelConstructor
 
 222          preReuse : Internal use
 
 223          preFlat : Internal use
 
 224  
 
 225          RETURNS: getGenerator method returns a Generator of the specified class
 
 226          """ 
 227          self.mspec = mspec 
 228          # user events are additional to the intrinsic constraint events
 
 229          # that are made automatically from the variables' bounds information
 
 230          if userevents is None: 
 231              self.userevents = [] 
 232          else: 
 233              self.userevents = copy.copy(userevents) 
 234          if userfns is None: 
 235              self.userfns = {} 
 236          else: 
 237              # ensure a list of Symbolic defs is converted to
 
 238              # the dictionary of string signatures and definitions format
 
 239              self.userfns = Symbolic.ensureStrArgDict(copy.copy(userfns)) 
 240          self.unravelInfo = unravelInfo   # presently just a Boolean 
 241          if isinstance(targetGen, str): 
 242              self.targetGen = targetGen 
 243          else: 
 244              raise TypeError("targetGen argument must be a string") 
 245          if algparams is None: 
 246              self.algparams = {} 
 247          else: 
 248              self.algparams = copy.copy(algparams) 
 249          self.indepvarname = indepvar[0] 
 250          self.indepvardomain = indepvar[1] 
 251          self.tdata = tdata 
 252          if inputs is None: 
 253              self.inputs = {} 
 254          else: 
 255              self.inputs = copy.copy(inputs) 
 256          if parvalues is None: 
 257              self.parvalues = {} 
 258          else: 
 259              self.parvalues = copy.copy(parvalues) 
 260          if icvalues is None: 
 261              self.icvalues = {} 
 262          else: 
 263              self.icvalues = copy.copy(icvalues) 
 264          self.checklevel = checklevel 
 265          self.forcedAuxVars = [] 
 266          if options is None: 
 267              self.optDict = {} 
 268          else: 
 269              self.optDict = copy.copy(options) 
 270          if reuseTerms is None: 
 271              self.reuseTerms = {} 
 272          else: 
 273              self.reuseTerms = copy.copy(reuseTerms) 
 274          self.vfcodeinsert_start = "" 
 275          self.vfcodeinsert_end = "" 
 276          if activatedBounds is None: 
 277              self.activatedBounds = {} 
 278          else: 
 279              self.activatedBounds = copy.copy(activatedBounds) 
 280          self.activateAllBounds = activateAllBounds  # overrides activatedBounds 
 281          if abseps is None: 
 282              self.abseps = 1e-13 
 283          else: 
 284              self.abseps = abseps 
 285          # List of parameter names associated solely with events
 
 286          if eventPars is None: 
 287              self.eventPars = [] 
 288          else: 
 289              self.eventPars = copy.copy(eventPars) 
 290          self.preReuse = preReuse 
 291          if preReuseTerms is None: 
 292              self.preReuseTerms = {} 
 293          else: 
 294              self.preReuseTerms = copy.copy(preReuseTerms) 
 295          self.preFlat = preFlat

 296  
 
 297  
 


298 -    def setForcedAuxVars(self, vlist):


299          self.forcedAuxVars = vlist

 300  
 


301 -    def setReuseTerms(self, rdict):


302          self.reuseTerms = rdict 
 303          self.preReuse = False

 304  
 


305 -    def setVfCodeInsertStart(self, codestr):


306          self.vfcodeinsert_start = codestr

 307  
 


308 -    def setVfCodeInsertEnd(self, codestr):


309          self.vfcodeinsert_end = codestr

 310  
 


311 -    def setOptions(self, optDict):


312          # e.g. for 'nobuild' option for C-based ODE integrators
 
 313          self.optDict = optDict

 314  
 


315 -    def addEvents(self, evtarg, eventPars=None):


316          if isinstance(evtarg, list): 
 317              self.userevents.extend(evtarg) 
 318          elif isinstance(evtarg, Events.Event): 
 319              self.userevents.append(evtarg) 
 320          else: 
 321              raise TypeError("Invalid event or event list") 
 322          # Use this list to determine whether parameters are event specific
 
 323          if eventPars is not None and eventPars != [] and eventPars != '': 
 324              if isinstance(eventPars, list): 
 325                  self.eventPars.extend(eventPars) 
 326              elif isinstance(eventPars, str): 
 327                  self.eventPars.append(eventPars)

 328  
 
 329  
 


330 -    def addFunctions(self, fnarg):


331          self.userfns.update(Symbolic.ensureStrArgDict(copy.copy(fnarg)))

 332  
 


333 -    def activateBounds(self, varname=None, which_bounds='all'):


334          """which_bounds argument is either 'lo', 'hi', or a pair ('lo', 'hi').
 
 335          Calling with no arguments activates all bounds.""" 
 336          if varname is None and which_bounds=='all': 
 337              self.activateAllBounds = True 
 338          else: 
 339              entry = [False,False] 
 340              if 'hi' in which_bounds: 
 341                  entry[1] = True 
 342              if 'lo' in which_bounds: 
 343                  entry[0] = True 
 344              self.activatedBounds[varname] = entry

 345  
 
 346  
 


347 -    def getGenerator(self):


348          """Build and return a Generator instance from an abstract
 
 349          specification.""" 
 350          ### Instantiate (flatten) target model structured specification
 
 351          ## Flatten ModelSpec self.mspec using indepvarname global and inputs
 
 352          # and using connectivity bindings (latter not yet implemented)
 
 353          globalRefs = [self.indepvarname] + self.inputs.keys() 
 354          self.mspec.eventPars = copy.copy(self.eventPars) 
 355  
 
 356          if not self.preFlat: 
 357              try: 
 358                  flatspec = self.mspec.flattenSpec(multiDefUnravel=self.unravelInfo,
 
 359                                                    globalRefs=globalRefs,
 
 360                                                    ignoreInputs=True) 
 361              except KeyboardInterrupt: 
 362                  raise 
 363              except: 
 364                  print "Problem flattening Model Spec '%s'"%self.mspec.name 
 365                  print "Global refs: ", globalRefs 
 366                  raise 
 367          else: 
 368              flatspec = self.mspec.flatSpec 
 369  
 
 370          FScompatibleNames = flatspec['FScompatibleNames'] 
 371          FScompatibleNamesInv = flatspec['FScompatibleNamesInv'] 
 372          ## Check target Generator info
 
 373          if self.targetGen in self.mspec.compatibleGens \
 
 374                     and self.targetGen in Generator.theGenSpecHelper: 
 375              gsh = Generator.theGenSpecHelper(self.targetGen) 
 376              if gsh.lang not in self.mspec.targetLangs: 
 377                  raise ValueError("Incompatible target language between supplied"
 
 378                                   " ModelSpec and target Generator") 
 379          else: 
 380              print "ModelSpec's compatible Generators:", \
 
 381                    ", ".join(self.mspec.compatibleGens) 
 382              print "ModelConstructor target Generator:", self.targetGen 
 383              raise ValueError("Target Generator mismatch during generator "
 
 384                               "construction") 
 385          self.targetLang = gsh.lang 
 386          ## Make Generator initialization argument dictionary
 
 387          a = args(abseps=self.abseps) 
 388          a.pars = {} 
 389          parnames = flatspec['pars'].keys() 
 390          for p, valstr in flatspec['pars'].iteritems(): 
 391              if valstr == '': 
 392                  if FScompatibleNamesInv(p) not in self.parvalues: 
 393                      raise ValueError("Parameter %s is missing a value"%FScompatibleNamesInv(p)) 
 394              else: 
 395                  if valstr == p: 
 396                      # placeholder
 
 397                      a.pars[p] = 0 
 398                      #raise NotImplementedError
 
 399                  else: 
 400                      try: 
 401                          a.pars[p] = float(valstr) 
 402                      except ValueError: 
 403                          raise ValueError("Invalid parameter value set in ModelSpec"
 
 404                                           " for '%s', value: %s"%(p,valstr)) 
 405          # override any par vals set in ModelSpec with those explicitly set
 
 406          # here
 
 407          for p, val in self.parvalues.iteritems(): 
 408              try: 
 409                  pr = FScompatibleNames(p) 
 410              except KeyError: 
 411                  raise NameError("Parameter '%s' missing from ModelSpec"%p) 
 412              if pr not in flatspec['pars']: 
 413                  raise NameError("Parameter '%s' missing from ModelSpec"%p) 
 414              a.pars[pr] = val 
 415          if self.icvalues != {}: 
 416              a.ics = {} 
 417              for v, val in self.icvalues.iteritems(): 
 418                  try: 
 419                      vr = FScompatibleNames(v) 
 420                  except KeyError: 
 421                      raise NameError("Variable '%s' missing from ModelSpec"%v) 
 422                  if vr not in flatspec['vars']: 
 423                      raise NameError("Variable '%s' missing from ModelSpec"%v) 
 424                  a.ics[vr] = val 
 425          a.tdomain = self.indepvardomain 
 426          if self.tdata is not None: 
 427              a.tdata = self.tdata 
 428          # a.ttype = float or int ?
 
 429          a.inputs = self.inputs 
 430          a.name = self.mspec.name 
 431          xdomain = {} 
 432          xtype = {} 
 433          pdomain = {} 
 434          for k, d in flatspec['domains'].iteritems(): 
 435              # e.g. d == (float, Continuous, [-Inf, Inf])
 
 436              if k in flatspec['vars']: 
 437                  if isinstance(d[2], _num_types): 
 438                      xdomain[k] = d[2] 
 439                  elif len(d[2]) == 2: 
 440                      xdomain[k] = d[2] 
 441                  else: 
 442                      raise ValueError("Domain spec must be a valid interval") 
 443                  xtype[k] = d[0] 
 444              elif k in flatspec['pars']: 
 445                  assert len(d[2]) == 2, "Domain spec must be a valid interval" 
 446                  pdomain[k] = d[2] 
 447              # Eliminate this test because it won't work for hybrid models, where an indicator
 
 448              # variable is needed, which is set as a discrete variable with integer value
 
 449              #if d[1] != gsh.domain:
 
 450              #    raise AssertionError("Domain mismatch (%s) with target Generator's (%s)"%(d[1],gsh.domain))
 
 451          a.xdomain = xdomain 
 452          a.pdomain = pdomain 
 453          a.xtype = xtype 
 454          exp_vars = [v for (v,t) in flatspec['spectypes'].items() \
 
 455                           if t == 'ExpFuncSpec'] 
 456          rhs_vars = [v for (v,t) in flatspec['spectypes'].items() \
 
 457                           if t == 'RHSfuncSpec'] 
 458          imp_vars = [v for (v,t) in flatspec['spectypes'].items() \
 
 459                           if t == 'ImpFuncSpec'] 
 460          if gsh.specType == 'RHSfuncSpec': 
 461              assert imp_vars == [], "Cannot use implicitly defined variables" 
 462   #           assert self.forcedAuxVars == [], "Cannot force auxiliary variables"
 
 463              varnames = rhs_vars 
 464              auxvarnames = exp_vars 
 465          elif gsh.specType == 'ExpFuncSpec': 
 466              assert imp_vars == [], "Cannot use implicitly defined variables" 
 467              assert rhs_vars == [], "Cannot use RHS-type variables" 
 468              varnames = exp_vars 
 469              invalid_auxvars = remain(self.forcedAuxVars, varnames) 
 470              if invalid_auxvars == []: 
 471                  # then all forced aux varnames were legitimate
 
 472                  # so remove them from varnames and put them in auxvarnames
 
 473                  varnames = remain(varnames, self.forcedAuxVars) 
 474                  auxvarnames = self.forcedAuxVars 
 475              else: 
 476                  print "Invalid auxiliary variable names:" 
 477                  print invalid_auxvars 
 478  #               raise ValueError("Forced auxiliary variable names were invalid")
 
 479          elif gsh.specType == 'ImpFuncSpec': 
 480              assert rhs_vars == [], "Cannot use RHS-type variables" 
 481              varnames = imp_vars 
 482              auxvarnames = exp_vars 
 483          # search for explicit variable interdependencies and resolve by
 
 484          # creating 'reuseterms' declarations, substituting in the cross-ref'd
 
 485          # definitions
 
 486          # e.g. state variables v and w, and explicit aux vars are given by:
 
 487          #     x = 1+v
 
 488          #     y = f(x) + w
 
 489          # Here, y illegally depends on x, so define a 'reused' temporary
 
 490          # definition, and re-write in terms of that:
 
 491          #     temp = 1+v
 
 492          #     x = temp
 
 493          #     y = f(temp) + w
 
 494          # e.g. state variables v and w, aux var x:
 
 495          #     v' = 1-v -f(x)
 
 496          # Here, v illegally uses an auxiliary variable on the RHS, so make
 
 497          # a 'reused' substitution as before
 
 498          #
 
 499          # first pass to find which substitutions are needed
 
 500  
 
 501          # DO THIS PART AGAIN ONLY IF BOOLEAN IS SET
 
 502          if not self.preReuse: 
 503              reuseTerms, subsExpr = processReused(varnames+auxvarnames, auxvarnames,
 
 504                                                   flatspec, self.mspec._registry,
 
 505                                                   FScompatibleNames, FScompatibleNamesInv) 
 506  
 
 507              clash_reused = intersect(reuseTerms.keys(), self.reuseTerms.keys()) 
 508              if clash_reused != []: 
 509                  print "Clashing terms:", clash_reused 
 510                  raise ValueError("User-supplied reused terms clash with auto-"
 
 511                                   "generated terms") 
 512              # second pass, this time to actually make the substitutions
 
 513              for v in subsExpr: 
 514                  flatspec['vars'][v] = subsExpr[v] 
 515              reuseTerms.update(self.reuseTerms) 
 516              a.reuseterms = reuseTerms 
 517  
 
 518          else: 
 519              a.reuseterms = self.preReuseTerms 
 520  
 
 521          a.varspecs = dict(zip(varnames+auxvarnames, [flatspec['vars'][v] \
 
 522                                        for v in varnames+auxvarnames])) 
 523          a.auxvars = auxvarnames 
 524          a.fnspecs = self.userfns 
 525          try: 
 526              a.fnspecs.update(flatspec['auxfns']) 
 527          except KeyError: 
 528              # no aux fns defined in flat spec!
 
 529              pass 
 530          a.checklevel = self.checklevel 
 531          a.algparams = self.algparams 
 532          if self.vfcodeinsert_start != "": 
 533              a.vfcodeinsert_start = self.vfcodeinsert_start 
 534          if self.vfcodeinsert_end != "": 
 535              a.vfcodeinsert_end = self.vfcodeinsert_end 
 536          ## Events
 
 537          events = [] 
 538          # make events from bound constraints (activated accordingly)
 
 539          # (parameter bounds only useful for continuation with PyCont)
 
 540          nonEvtParnames = remain(parnames, self.eventPars) 
 541          domnames = varnames+nonEvtParnames 
 542          for xname in domnames: 
 543              hier_name_lo = FScompatibleNamesInv(xname)+"_domlo" 
 544              FScompatibleNames[hier_name_lo] = xname+"_domlo" 
 545              FScompatibleNamesInv[xname+"_domlo"] = hier_name_lo 
 546              hier_name_hi = FScompatibleNamesInv(xname)+"_domhi" 
 547              FScompatibleNames[hier_name_hi] = xname+"_domhi" 
 548              FScompatibleNamesInv[xname+"_domhi"] = hier_name_hi 
 549          if self.activateAllBounds: 
 550              a.activatedbounds = {}.fromkeys(domnames,(True,True)) 
 551          else: 
 552              a.activatedbounds = self.activatedBounds 
 553          a.enforcebounds = True  # not currently manipulated, used in Generator baseclasses 
 554          # add events from user events
 
 555          for e in self.userevents: 
 556              if e not in events: 
 557                  events.append(e) 
 558              else: 
 559                  raise ValueError("Repeated event definition!") 
 560  
 
 561          a.events = events 
 562          # Add any additional special options (e.g. 'nobuild' directive)
 
 563          for k,v in self.optDict.iteritems(): 
 564              if hasattr(a, k): 
 565                  raise KeyError("'%s' already exists as a Generator argument"%k) 
 566              a.k = v 
 567          a.FScompatibleNames = FScompatibleNames 
 568          a.FScompatibleNamesInv = FScompatibleNamesInv 
 569  
 
 570          # Parameters solely associated with events -- don't make domain events for them
 
 571          a.eventPars = self.eventPars 
 572  
 
 573          # keep a copy of the arguments in self for users to see what was done
 
 574          self.conargs = copy.copy(a) 
 575          # TEMP FOR DEBUGGING VECTOR FIELD
 
 576  #        a['vfcodeinsert_end'] = '    print xnew0, xnew1, xnew2, xnew3, xnew4, xnew5\n'
 
 577  #        a['vfcodeinsert_start'] = '    print t\n    print x\n    print parsinps\n    import sys; sys.stdout.flush()'
 
 578  
 
 579          ## Make Generator
 
 580          try: 
 581              return gsh.genClass(args(**filteredDict(a.__dict__,
 
 582                              gsh.genClass._needKeys+gsh.genClass._optionalKeys))) 
 583          except: 
 584              print "Problem initializing target Generator '%s'"%self.targetGen 
 585              raise

 586  
 
 587  
 


588 -    def __del__(self):


589          del self.userevents 
 590          if hasattr(self, 'conargs'): 
 591              try: 
 592                  del self.conargs.events 
 593              except AttributeError: 
 594                  pass

 595  
 
 596  
 
 597  # -----------------------------------------------------------------------------
 
 598  
 
 599  
 


600 -class ModelConstructor(object):


601 -    def __init__(self, name, userevents=None, userfns=None, unravelInfo=True,
 
 602                   inputs=None, checklevel=2, activateAllBounds=False,
 
 603                   generatorspecs=None, indepvar=('t',[-Inf,Inf]),
 
 604                   parvalues=None, icvalues=None, tdata=None, reuseTerms=None,
 
 605                   withJac=None, withJacP=None, featureDicts=None,
 
 606                   abseps=None, eventtol=None, eventPars=None,
 
 607                   withStdEvts=None, stdEvtArgs=None):


608          """Notes for initialization arguments.
 
 609  
 
 610          name : string name of this ModelConstructor
 
 611  
 
 612          ** The following are applied to all Generators
 
 613  
 
 614          activateAllBounds : Boolean
 
 615          checklevel : integer
 
 616          indepvar : pair of (independent var name, pair giving domain interval)
 
 617  
 
 618          ** The following are dictionaries keyed by Generator name, with values:
 
 619  
 
 620          generatorspecs : ModelSpecs
 
 621          userevents : list of Event objects
 
 622          userfns : dictionary of named user functions specs
 
 623          inputs : dictionary of Variable objects
 
 624          algparams : dictionary of algorithmic parameters for Generator
 
 625          parvalues : dictionary of parameter values
 
 626          icvalues : dictionary of initial condition values
 
 627          reuseterms : dictionary of reused terms in specifications
 
 628          eventPars : list of parameter names associated solely with events
 
 629          withStdEvts : Boolean for making standard events (bounds & turning points)
 
 630          stdEvtArgs : arguments for the standard events
 
 631          featureDicts : dictionary of Features for making each Model Interface
 
 632          withJac : Boolean for making Jacobian
 
 633          withJacP : Boolean for making Jacobian with respect to parameters
 
 634  
 
 635          RETURNS: Nothing, but getModel method returns an instantiated Model
 
 636            object when the specifications are complete and consistent.
 
 637          """ 
 638          self.name = name 
 639          self.forcedIntVars = [] 
 640          if generatorspecs is None: 
 641              self._generators = {} 
 642          else: 
 643              self._generators = copy.copy(generatorspecs) 
 644          if userevents is None: 
 645              self._events = {} 
 646          else: 
 647              self._events = copy.copy(userevents) 
 648          if userfns is None: 
 649              self._funcs = {} 
 650          else: 
 651              self._funcs = copy.copy(userfns) 
 652          self.indepvar = indepvar 
 653          self.indepvarname = self.indepvar[0] 
 654          self.eventmaps = {} 
 655          if reuseTerms is None: 
 656              self.reuseTerms = {} 
 657          else: 
 658              self.reuseTerms = copy.copy(reuseTerms) 
 659          if inputs is None: 
 660              self.inputs = {} 
 661          else: 
 662              self.inputs = copy.copy(inputs) 
 663          if parvalues is None: 
 664              self.parvalues = {} 
 665          else: 
 666              self.parvalues = copy.copy(parvalues) 
 667          if icvalues is None: 
 668              self.icvalues = {} 
 669          else: 
 670              self.icvalues = copy.copy(icvalues) 
 671          self.tdata = tdata 
 672          self.checklevel = checklevel 
 673          self.unravelInfo = unravelInfo   # presently just a Boolean 
 674          self.activatedBounds = {} 
 675          self.activateAllBounds = activateAllBounds  # overrides activatedBounds 
 676          if abseps is None: 
 677              abseps = 1e-13 
 678          self.abseps = abseps 
 679          if eventtol is None: 
 680              eventtol = abseps * 1e3 
 681          self.eventtol = eventtol 
 682          if withJac is None: 
 683              self.withJac = {} 
 684          else: 
 685              self.withJac = copy.copy(withJac) 
 686          if withJacP is None: 
 687              self.withJacP = {} 
 688          else: 
 689              self.withJacP = copy.copy(withJacP) 
 690          if withStdEvts is None: 
 691              self.withStdEvts = {} 
 692          else: 
 693              self.withStdEvts = copy.copy(withStdEvts) 
 694          if stdEvtArgs is None: 
 695              self.stdEvtArgs = {} 
 696          else: 
 697              self.stdEvtArgs = copy.copy(stdEvtArgs) 
 698          if featureDicts is None: 
 699              self.featureDicts = {} 
 700          else: 
 701              self.featureDicts = copy.copy(featureDicts) 
 702  
 
 703          # At this point, process our reuse terms, so we don't have to do it
 
 704          # again. Includes flattening the spec?
 
 705          self.preFlat = {} # dictionary keyed by generator; whether it's been flattened already (change in addEvents) 
 706          self.preReuse = {} # dictionary keyed by generator; whether it's reused terms have been processed already 
 707          self.preReuseTerms = {} 
 708          for g in self._generators: 
 709              self.preFlat[g] = False 
 710              self.preReuse[g] = False 
 711              self.preReuseTerms[g] = {} 
 712  
 
 713          if self.withJac != {}: 
 714              self.createJac() 
 715          if self.withJacP != {}: 
 716              self.createJacP() 
 717  
 
 718          # dictionary of lists of parameter names associated solely with events;
 
 719          # keyed by generator name
 
 720          if eventPars is None: 
 721              self._eventPars = {} 
 722          else: 
 723              self._eventPars = copy.copy(eventPars) 
 724  
 
 725          if self.withStdEvts != {}: 
 726              self.preprocessFlatten() 
 727              self.preprocessReuseTerms() 
 728              self.createStdEvts()

 729  
 
 730  
 


731 -    def __repr__(self):


732          return "ModelConstructor %s"%self.name

 733  
 


734 -    def preprocessFlatten(self):


735          globalRefs = [self.indepvarname] + self.inputs.keys() 
 736          for g in self._generators: 
 737              gspec = self._generators[g]['modelspec'] 
 738              try: 
 739                  flatspec = gspec.flattenSpec(multiDefUnravel=self.unravelInfo, globalRefs=globalRefs,
 
 740                                               ignoreInputs=True) 
 741              except KeyboardInterrupt: 
 742                  raise 
 743              except: 
 744                  print "Problem flattening Model Spec %s"%self.mspec.name 
 745                  print "Global refs: ", globalRefs 
 746                  raise 
 747              self.preFlat[g] = True

 748  
 


749 -    def preprocessReuseTerms(self):


750          for g in self._generators: 
 751              gspec = self._generators[g]['modelspec'] 
 752              assert self.preFlat[g] 
 753              flatspec = gspec.flatSpec 
 754              gsh = Generator.theGenSpecHelper(self._generators[g]['target']) 
 755  
 
 756              FScompatibleNames = flatspec['FScompatibleNames'] 
 757              FScompatibleNamesInv = flatspec['FScompatibleNamesInv'] 
 758  
 
 759              exp_vars = [v for (v,t) in flatspec['spectypes'].items() \
 
 760                          if t == 'ExpFuncSpec'] 
 761              rhs_vars = [v for (v,t) in flatspec['spectypes'].items() \
 
 762                          if t == 'RHSfuncSpec'] 
 763              imp_vars = [v for (v,t) in flatspec['spectypes'].items() \
 
 764                          if t == 'ImpFuncSpec'] 
 765              if gsh.specType == 'RHSfuncSpec': 
 766                  assert imp_vars == [], "Cannot use implicitly defined variables" 
 767  #                assert self.forcedAuxVars == [], "Cannot force auxiliary variables"
 
 768                  varnames = rhs_vars 
 769                  auxvarnames = exp_vars 
 770              elif gsh.specType == 'ExpFuncSpec': 
 771                  assert imp_vars == [], "Cannot use implicitly defined variables" 
 772                  assert rhs_vars == [], "Cannot use RHS-type variables" 
 773                  varnames = exp_vars 
 774                  #invalid_auxvars = remain(self.forcedAuxVars, varnames)
 
 775                  #if invalid_auxvars == []:
 
 776                      ## then all forced aux varnames were legitimate
 
 777                      ## so remove them from varnames and put them in auxvarnames
 
 778                      #varnames = remain(varnames, self.forcedAuxVars)
 
 779                      #auxvarnames = self.forcedAuxVars
 
 780                  #else:
 
 781                      #print "Invalid auxiliary variable names:"
 
 782                      #print invalid_auxvars
 
 783                      #raise ValueError("Forced auxiliary variable names were invalid")
 
 784              elif gsh.specType == 'ImpFuncSpec': 
 785                  assert rhs_vars == [], "Cannot use RHS-type variables" 
 786                  varnames = imp_vars 
 787                  auxvarnames = exp_vars 
 788  
 
 789              reuseTerms, subsExpr = processReused(varnames+auxvarnames, auxvarnames,
 
 790                                                   flatspec, gspec._registry,
 
 791                                                   FScompatibleNames, FScompatibleNamesInv) 
 792  
 
 793              clash_reused = intersect(reuseTerms.keys(), self.reuseTerms.keys()) 
 794              if clash_reused != []: 
 795                  print "Clashing terms:", clash_reused 
 796                  raise ValueError("User-supplied reused terms clash with auto-"
 
 797                                   "generated terms") 
 798  
 
 799              # second pass, this time to actually make the substitutions
 
 800              for v in subsExpr: 
 801                  flatspec['vars'][v] = subsExpr[v] 
 802              reuseTerms.update(self.reuseTerms) 
 803  
 
 804              # Need to make reuseterms universally available
 
 805              self.preReuse[g] = True 
 806              self.preReuseTerms[g] = reuseTerms

 807  
 


808 -    def addModelInfo(self, genSpec, genTarg, genAlgPars={}, unravelInfo={},
 
 809                     genOpts={}):


810          """genSpec can either be a complete ModelSpec description or a
 
 811          string-based dictionary of definitions.
 
 812          """ 
 813          if isinstance(genSpec, dict): 
 814              genSpec = args(**genSpec) 
 815          if len(genAlgPars)==0: 
 816              # in case user gave a string-based definition, algparams
 
 817              # may already be given in that definition.
 
 818              if hasattr(genSpec, 'algparams'): 
 819                  genAlgPars = genSpec['algparams'] 
 820          if hasattr(genSpec, 'events'): 
 821              self.addEvents(genSpec.name, genSpec.events) 
 822          self._generators[genSpec.name] = args(modelspec=genSpec,
 
 823                                            target=genTarg,
 
 824                                            algparams=copy.copy(genAlgPars),
 
 825                                            unravelInfo=copy.copy(unravelInfo),
 
 826                                            options=copy.copy(genOpts))

 827  
 


828 -    def createStdEvts(self):


829          evtArgsDefaults =  {'eventtol': self.eventtol,
 
 830                              'eventdelay': self.eventtol*1e4,
 
 831                              'starttime': 0,
 
 832                              'term': False,
 
 833                              'active': False} 
 834  
 
 835          rhsEvtTypeList = ['val', 'deriv', 'stat'] 
 836          expEvtTypeList = ['val'] 
 837          withEvtParList = ['val', 'deriv'] 
 838          evtDirList = [('inc', 1), ('dec', -1), ('neut', 0)] 
 839          specList = ['auxfns', 'vars'] 
 840          evtParList = [] 
 841          for g in self._generators: 
 842              targetLang = Generator.theGenSpecHelper(self._generators[g]['target']).lang 
 843              evtList = [] 
 844              try: 
 845                  makeEvts = self.withStdEvts[g] 
 846              except KeyError: 
 847                  makeEvts = False 
 848              if makeEvts: 
 849                  gspec = self._generators[g]['modelspec'] 
 850                  if not self.preFlat[g]: 
 851                      print "Flattening" 
 852                      gspec.flattenSpec() 
 853                  fspec = gspec.flatSpec 
 854                  # name maps
 
 855                  FScNM = fspec['FScompatibleNames'] 
 856                  FScNMInv = fspec['FScompatibleNamesInv'] 
 857                  # temp dict to store new event par name mappings
 
 858                  FSc_update_dict = {} 
 859                  FScInv_update_dict = {} 
 860                  try: 
 861                      stdEvtArgs = self.stdEvtArgs[g] 
 862                  except KeyError: 
 863                      stdEvtArgs = evtArgsDefaults 
 864  
 
 865                  # Make event functions for auxfns
 
 866                  evtTypeList = expEvtTypeList 
 867                  for s in specList: 
 868                      if s not in fspec.keys(): 
 869                          continue 
 870  
 
 871                      # auxfns are only explicit types
 
 872                      if s == 'auxfns': 
 873                          evtTypeList = expEvtTypeList 
 874                          checkEvtType = False 
 875                      else: 
 876                          evtTypeList = [] 
 877                          checkEvtType = True 
 878  
 
 879                      for f in fspec[s].keys(): 
 880                          if checkEvtType: 
 881                              if fspec['spectypes'][f] == 'ExpFuncSpec': 
 882                                  evtTypeList = expEvtTypeList 
 883                              elif fspec['spectypes'][f] == 'RHSfuncSpec': 
 884                                  evtTypeList = rhsEvtTypeList 
 885                              else: 
 886                                  raise PyDSTool_ValueError("Don't know this "
 
 887                                                            "spec type.") 
 888  
 
 889                              # val, deriv, stat
 
 890                              for evtType in evtTypeList: 
 891                                  # inc, dec, neut
 
 892                                  for i in range(len(evtDirList)): 
 893                                      # make event, parameter names (auxfns can only hit values, not test derivs)
 
 894                                      evtName = f + '_'+ evtType + '_' + evtDirList[i][0] + '_evt' 
 895                                      evtNameFSInv = FScNMInv(f) + '_'+ evtType + '_' + evtDirList[i][0] + '_evt' 
 896                                      # If there is an event parameter associated with this kind of event
 
 897                                      if evtType in withEvtParList: 
 898                                          parname = evtName+'_p' 
 899                                          FScInv_update_dict[parname] = evtNameFSInv+'_p' 
 900                                          FSc_update_dict[evtNameFSInv+'_p'] = parname 
 901                                          # default param value is 0
 
 902                                          par = Symbolic.Par(str(0), parname) 
 903                                          par.compatibleGens = gspec.compatibleGens 
 904                                          # add parameter to modelspec pars
 
 905                                          # add parameters names, values to flattened spec
 
 906                                          gspec.pars[parname] = par 
 907                                          fspec['pars'][parname] = 0 # default value is 0 
 908  
 
 909                                      # make the associated event
 
 910                                      # Error correction: var val events are on the variable value, not the deriv. value
 
 911                                      if s == 'vars' and evtType == 'val': 
 912                                          evtStr = f + ' - '  + parname 
 913                                      elif evtType in withEvtParList: 
 914                                          evtStr = fspec[s][f] + '-' + parname 
 915                                      else: 
 916                                          evtStr = fspec[s][f] 
 917                                      # Adding the event is the same for all cases
 
 918                                      evtDir = evtDirList[i][1] 
 919                                      evtArgs = stdEvtArgs 
 920                                      evtArgs['name'] = evtName 
 921                                      evtSuccess = True 
 922                                      # Some events can't be made if they are ill-formed (currently arises
 
 923                                      # with the neural toolbox auxilliary variables)
 
 924                                      try: 
 925                                          if self.preReuse[g]: 
 926                                              # This has a conflict with LowLevelEvent class which expects
 
 927                                              # there to just be a return string -- fix later
 
 928                                              #       reuseterms = self.preReuseTerms[g]
 
 929                                              reuseterms = {} 
 930                                          else: 
 931                                              reuseterms = {} 
 932                                          theEvt = Events.makeZeroCrossEvent(expr=evtStr,
 
 933                                                                             dircode=evtDir,
 
 934                                                                             argDict=evtArgs,
 
 935                                                                             targetlang=targetLang,
 
 936                                                                             flatspec=fspec,
 
 937                                                                             reuseterms=reuseterms) 
 938                                      except ValueError, errinfo: 
 939                                          evtSuccess = False 
 940                                          #print "Warning: Could not make standard event " + evtName + " with definition " + evtStr
 
 941                                          #print "  Original problem: ", errinfo
 
 942                                          #print "  Skipping this event."
 
 943                                      if evtSuccess: 
 944                                          evtList.append(theEvt) 
 945                                      # Add the event parameter to evtParList even if building event was
 
 946                                      # a failure, since we have already made the parameter and added it to the
 
 947                                      # flatspec
 
 948                                      if evtType in withEvtParList: 
 949                                          evtParList.append(parname) 
 950  
 
 951              # Do something with the events that are made
 
 952              if evtList != []: 
 953                  self.addEvents(g, evtList) 
 954  
 
 955              # Do something with the event par lists
 
 956              if evtParList != []: 
 957                  # add event par name mappings
 
 958                  FScNM.update(FSc_update_dict) 
 959                  FScNMInv.update(FScInv_update_dict) 
 960                  if g in self._eventPars.keys(): 
 961                      self._eventPars[g].extend(evtParList) 
 962                  else: 
 963                      self._eventPars[g] = evtParList

 964  
 


965 -    def createJacP(self):


966          for g in self._generators: 
 967              if self.withJac[g]: 
 968                  gspec = self._generators[g]['modelspec'] 
 969                  # haven't made generator yet so don't know which are the
 
 970                  # regular RHS variables
 
 971                  candidate_vars = gspec.funcSpecDict['vars']  # Quantity objects 
 972                  vars = {} 
 973                  auxvars = {} 
 974                  for v in candidate_vars: 
 975                      vname = str(v).replace('.','_') 
 976                      if v.specType == 'RHSfuncSpec': 
 977                          vars[vname] = gspec.flatSpec['vars'][vname] 
 978                      elif v.specType == 'ExpFuncSpec': 
 979                          auxvars[vname] = gspec.flatSpec['vars'][vname] 
 980                  varnames = vars.keys() 
 981                  varnames.sort() 
 982                  # RHS specs may contain aux vars, so need to substitute their
 
 983                  # definitions from flatSpec
 
 984                  varspecs = {} 
 985                  for vn in varnames: 
 986                      q = ModelSpec.QuantSpec('__temp__', vars[vn]) 
 987                      varspecs[vn] = str(q.eval(auxvars)) 
 988  
 
 989                  # Find parameters with w.r.t which to take derivs
 
 990                  candidate_pars = gspec.funcSpecDict['pars']  # Quantity objects 
 991                  parnames = [] 
 992  
 
 993                  try: 
 994                      evtPars = self._eventPars[g] 
 995                  except KeyError: 
 996                      evtPars = [] 
 997  
 
 998                  for p in candidate_pars: 
 999                      pname_with_dot = str(p) 
1000                      pname_no_dot = str(p).replace('.','_') 
1001                      if pname_with_dot in evtPars or pname_no_dot in evtPars: 
1002                          pass 
1003                      else: 
1004                          parnames.append(pname_no_dot) 
1005                  parnames.sort() 
1006  
 
1007                  jacP = Symbolic.Diff([varspecs[vn] for vn in varnames],
 
1008                             parnames).renderForCode() 
1009                  self.addFunctions(g, Symbolic.Fun(jacP, ['t'] + varnames,
 
1010                                                    'Jacobian_pars'))


1011 -    def createJac(self):


1012          for g in self._generators: 
1013              if self.withJac[g]: 
1014                  gspec = self._generators[g].modelspec 
1015                  # haven't made generator yet so don't know which are the
 
1016                  # regular RHS variables
 
1017                  candidate_vars = gspec.funcSpecDict['vars']  # Quantity objects 
1018                  vars = {} 
1019                  auxvars = {} 
1020                  for v in candidate_vars: 
1021                      vname = str(v).replace('.','_') 
1022                      if v.specType == 'RHSfuncSpec': 
1023                          vars[vname] = gspec.flatSpec['vars'][vname] 
1024                      elif v.specType == 'ExpFuncSpec': 
1025                          auxvars[vname] = gspec.flatSpec['vars'][vname] 
1026                  varnames = vars.keys() 
1027                  varnames.sort() 
1028                  # RHS specs may contain aux vars, so need to substitute their
 
1029                  # definitions from flatSpec
 
1030                  varspecs = {} 
1031                  for vn in varnames: 
1032                      q = ModelSpec.QuantSpec('__temp__', vars[vn]) 
1033                      varspecs[vn] = str(q.eval(auxvars)) 
1034                  jac = Symbolic.Diff([varspecs[vn] for vn in varnames],
 
1035                             varnames).renderForCode() 
1036                  self.addFunctions(g, Symbolic.Fun(jac, ['t'] + varnames,
 
1037                                                    'Jacobian'))

1038  
 


1039 -    def createGenerators(self):


1040          """Create the generators from the source specs, either in the form
 
1041          of dicts or args objects, or as a GDescriptor.
 
1042  
 
1043          Still some teething trouble getting expected types neat and tidy.
 
1044          """ 
1045          # 1. build constituent generators from whichever source
 
1046          # 2. combine all generators' FScompatibleNames symbol maps
 
1047          FScompatibleNames = {} 
1048          FScompatibleNamesInv = {} 
1049          genObjs = {} 
1050          assert len(self._generators) > 0, "No Generator descriptions found" 
1051          for gname, geninfo in self._generators.iteritems(): 
1052              if isinstance(geninfo, args): 
1053                  if isinstance(geninfo.modelspec, args): 
1054                      # assume geninfo is traditional string definition
 
1055                      gen = self._genFromStrings(geninfo) 
1056                  else: 
1057                      # convert ModelSpec to GDescriptor
 
1058                      gen = self._genFromMSpec(GDescriptor(**geninfo.__dict__)) 
1059              elif isinstance(geninfo, dict): 
1060                  gen = self._genFromMSpec(GDescriptor(**geninfo)) 
1061              else: 
1062                  # GDescriptor already
 
1063                  gen = self._genFromMSpec(geninfo) 
1064              if gname != gen.name: 
1065                  print gname, " vs.", gen.name 
1066                  raise ValueError("Generator name mismatch in gen descriptor") 
1067              genObjs[gen.name] = gen 
1068              # assume that there won't be any name clashes (there shouldn't be)
 
1069              FScompatibleNames.update(gen._FScompatibleNames.lookupDict) 
1070              FScompatibleNamesInv.update(gen._FScompatibleNamesInv.lookupDict) 
1071          return genObjs, genObjs.keys(), FScompatibleNames, FScompatibleNamesInv

1072  
 


1073 -    def _genFromStrings(self, geninfodesc):


1074          genStrings = geninfodesc['modelspec'] 
1075          # don't include event-related info in attrs because it's used
 
1076          # for event mappings
 
1077          attrs = [self.preReuse, self.preReuseTerms, self._funcs,
 
1078                   self.preFlat, self.parvalues, self.icvalues] 
1079          if sometrue([len(a) > 0 for a in attrs]): 
1080              raise ValueError("Can't mix string-based generator specs "
 
1081                               "with spec info added to ModelConstructor "
 
1082                               "object") 
1083          gsh = Generator.theGenSpecHelper(geninfodesc['target']) 
1084          return gsh.genClass(genStrings)

1085  
 


1086 -    def _genFromMSpec(self, geninfodesc):


1087          genSpec = geninfodesc.modelspec 
1088          genTarg = geninfodesc.target 
1089          genAlgPars = geninfodesc.algparams 
1090          if self.inputs is not None: 
1091              genInputs = self.inputs 
1092          else: 
1093              genInputs = {} 
1094          genUnravelInfo = geninfodesc.unravelInfo 
1095          genOpts = geninfodesc.options 
1096          try: 
1097              genEvents = self._events[genSpec.name] 
1098          except KeyError: 
1099              genEvents = [] 
1100          else: 
1101              if not isinstance(genEvents, list): 
1102                  genEvents = [genEvents] 
1103          try: 
1104              genEventPars = self._eventPars[genSpec.name] 
1105          except KeyError: 
1106              genEventPars = [] 
1107          else: 
1108              if not isinstance(genEventPars, list): 
1109                  genEventPars = [genEventPars] 
1110  
 
1111          try: 
1112              genFns = self._funcs[genSpec.name] 
1113          except KeyError: 
1114              genFns = None 
1115          try: 
1116              preReuse = self.preReuse[genSpec.name] 
1117          except KeyError: 
1118              self.preReuse[genSpec.name] = False 
1119              preReuse = False 
1120          try: 
1121              preReuseTerms = self.preReuseTerms[genSpec.name] 
1122          except KeyError: 
1123              self.preReuseTerms[genSpec.name] = {} 
1124              preReuseTerms = {} 
1125          try: 
1126              preFlat = self.preFlat[genSpec.name] 
1127          except KeyError: 
1128              self.preFlat[genSpec.name] = False 
1129              preFlat = False 
1130  
 
1131          # extract par values and ic values relevant to this generator
 
1132          genPars = {} 
1133          for p, val in self.parvalues.iteritems(): 
1134              # don't bother to check that p is a valid param name
 
1135              # for this generator -- that will be checked by
 
1136              # GeneratorConstructor
 
1137              if p in genSpec._registry: 
1138                  genPars[p] = val 
1139          genICs = {} 
1140          for v, val in self.icvalues.iteritems(): 
1141              # don't bother to check that v is a valid variable name
 
1142              # for this generator -- that will be checked by
 
1143              # GeneratorConstructor
 
1144              if v in genSpec._registry: 
1145                  genICs[v] = val 
1146          genCon = GeneratorConstructor(genSpec, checklevel=self.checklevel,
 
1147                                        userevents=genEvents,
 
1148                                        userfns=genFns,
 
1149                                        targetGen=genTarg,
 
1150                                        algparams=genAlgPars,
 
1151                                        tdata=self.tdata,
 
1152                                        indepvar=self.indepvar,
 
1153                                        parvalues=genPars,
 
1154                                        inputs=genInputs,
 
1155                                        icvalues=genICs,
 
1156                                        options=genOpts,
 
1157                                        unravelInfo=genUnravelInfo,
 
1158                                        reuseTerms=self.reuseTerms,
 
1159                                        abseps=self.abseps,
 
1160                                        activateAllBounds=self.activateAllBounds,
 
1161                                        activatedBounds=self.activatedBounds,
 
1162                                        eventPars=genEventPars,
 
1163                                        preReuse=preReuse,
 
1164                                        preReuseTerms=preReuseTerms,
 
1165                                        preFlat=preFlat) 
1166          return genCon.getGenerator()

1167  
 


1168 -    def getModel(self):


1169          """Build and return (hybrid) model made up of declared Generators and
 
1170          the mappings between events used to change vector fields in a hybrid
 
1171          system.
 
1172          """ 
1173          # 1. create generators
 
1174          genObjs, allGenNames, FScompatibleNames, FScompatibleNamesInv \
 
1175                     = self.createGenerators() 
1176          # 2. build event mappings
 
1177          modelInfoEntries = {} 
1178          modelInterfaces = {} 
1179          allModelNames = allGenNames 
1180          # TO DO: implement global consistency conditions
 
1181  
 
1182          # hack to allow test trajectories for one-gen models to avoid needing
 
1183          # pre-computation in order to test a trivial condition
 
1184          test_trajs = {} 
1185          for genname, gen in genObjs.iteritems(): 
1186              test_trajs[genname] = None 
1187          if len(genObjs)==1: 
1188              # singleton generator may need non-hybrid Model class unless
 
1189              # it contains discrete event state changes that map to itself
 
1190              useMI = False  # initial value 
1191              genname = genObjs.keys()[0] 
1192              if genname in self.eventmaps: 
1193                  for emap in self.eventmaps[genname]: 
1194                      if emap[1] != 'terminate': 
1195                          # then needs a hybrid model class
 
1196                          useMI = True 
1197                          break 
1198              if useMI and genname not in self.featureDicts: 
1199                  # then user didn't provide a feature to make a
 
1200                  # condition from. need to fill a default one in
 
1201                  # (simple, because there's only one model)
 
1202                  self.featureDicts = {genname: {MProject.always_feature('always'): True}} 
1203                  # 1 is not None so GenInterface._ensure_test_traj()
 
1204                  # will think that a test traj has already been computed
 
1205                  test_trajs[genname] = 1 
1206          else: 
1207              useMI = True 
1208          for hostGen, genObj in genObjs.iteritems(): 
1209              if useMI: 
1210                  m = embed(genObj,
 
1211                        tdata=genObj.indepvariable.depdomain.get()) 
1212                  try: 
1213                      DSi = MProject.intModelInterface(m,
 
1214                                  MProject.condition(self.featureDicts[hostGen]),
 
1215                                  test_traj=test_trajs[hostGen]) 
1216                  except KeyError: 
1217                      # no corresponding features to use
 
1218                      DSi = MProject.intModelInterface(m) 
1219                  allDSnames = allModelNames 
1220              else: 
1221                  DSi = MProject.GeneratorInterface(genObj) 
1222                  allDSnames = allGenNames 
1223              allGenTermEvents = genObj.eventstruct.getTermEvents() 
1224              allGenTermEvNames = [e[0] for e in allGenTermEvents] 
1225              if hostGen in self.eventmaps: 
1226                  genMaps = self.eventmaps[hostGen] 
1227                  genMapNames = [] 
1228                  for gmix, gmtuple in enumerate(genMaps): 
1229                      genMapNames.append(gmtuple[0]) 
1230                      if isinstance(gmtuple[1], tuple): 
1231                          # make mapping use model name
 
1232                          genMaps[gmix] = (gmtuple[0],
 
1233                                           (gmtuple[1][0],
 
1234                                            gmtuple[1][1])) 
1235                  for evname in remain(allGenTermEvNames, genMapNames): 
1236                      genMaps.append((evname, 'terminate')) 
1237                  modelInfoEntries[hostGen] = makeModelInfoEntry(DSi,
 
1238                                                             allDSnames,
 
1239                                                             genMaps) 
1240              else: 
1241                  # default for a generator without an event mapping is to
 
1242                  # terminate when its time is up.
 
1243                  genMaps = [('time', 'terminate')] 
1244                  for evname in allGenTermEvNames: 
1245                      genMaps.append((evname, 'terminate')) 
1246                  if not isfinite(genObj.indepvariable.depdomain[1]): 
1247                      print "Warning: Generator %s has no termination event"%genObj.name 
1248                      print "because it has an non-finite end computation time..." 
1249                  modelInfoEntries[hostGen] = makeModelInfoEntry(DSi,
 
1250                                                             allDSnames,
 
1251                                                             genMaps) 
1252          modelInfoDict = makeModelInfo(modelInfoEntries.values()) 
1253          # 3. build model
 
1254          mod_args = {'name': self.name,
 
1255                      'modelInfo': modelInfoDict,
 
1256                      'mspecdict': copy.copy(self._generators),
 
1257                      'eventPars': copy.copy(self._eventPars)} 
1258          if self.tdata is not None: 
1259              mod_args['tdata'] = self.tdata 
1260          if useMI: 
1261              model = Model.HybridModel(mod_args) 
1262          else: 
1263              model = Model.NonHybridModel(mod_args) 
1264          if self.forcedIntVars != []: 
1265              model.forceIntVars(self.forcedIntVars) 
1266          if self.icvalues != {}: 
1267              model.set(ics=self.icvalues) 
1268          if self.parvalues != {}: 
1269              model.set(pars=self.parvalues) 
1270          del genObjs 
1271          del modelInfoEntries 
1272          del modelInfoDict 
1273          return model

1274  
 


1275 -    def addFeatures(self, hostGen, featDict):


1276          """Update with feature -> Bool mapping dictionaries
 
1277          for a host generator.
 
1278          """ 
1279          if hostGen not in self.featureDicts: 
1280              self.featureDicts[hostGen] = {} 
1281          if isinstance(featDict, dict): 
1282              self.featureDicts[hostGen].update(featDict) 
1283          else: 
1284              raise TypeError("Invalid feature dictionary")

1285  
 


1286 -    def addEvents(self, hostGen, evTarg, eventPars=None):


1287          if hostGen not in self._events: 
1288              self._events[hostGen] = [] 
1289          if hostGen not in self._eventPars: 
1290              self._eventPars[hostGen] = [] 
1291          if isinstance(evTarg, (list, tuple)): 
1292              self._events[hostGen].extend(evTarg) 
1293          elif isinstance(evTarg, Events.Event): 
1294              self._events[hostGen].append(evTarg) 
1295          else: 
1296              raise TypeError("Invalid event or event list") 
1297          # Use this list to determine whether parameters are event specific
 
1298          if eventPars is not None and eventPars != [] and eventPars != '': 
1299              if isinstance(eventPars, list): 
1300                  self._eventPars[hostGen].extend(eventPars) 
1301              elif isinstance(eventPars, str): 
1302                  self._eventPars[hostGen].append(eventPars) 
1303              self._generators[hostGen].addEvtPars(eventPars)

1304  
 
1305  
 


1306 -    def addFunctions(self, hostGen, fnTarg):


1307          if hostGen not in self._funcs: 
1308              self._funcs[hostGen] = [] 
1309          if isinstance(fnTarg, list): 
1310              self._funcs[hostGen].extend(fnTarg) 
1311          elif isinstance(fnTarg, dict): 
1312              # for compatibility with list style of _funcs for symbolic Fun
 
1313              # objects, convert the string defs to symbolic form
 
1314              for k, v in fnTarg.items(): 
1315                  self._funcs[hostGen].append(Symbolic.Fun(v[1], v[0], k)) 
1316          else: 
1317              self._funcs[hostGen].append(fnTarg)

1318  
 
1319  
 


1320 -    def setReuseTerms(self, rdict):


1321          self.reuseTerms = rdict 
1322          for g in self._generators: 
1323              self.preReuse[g] = False

1324  
 


1325 -    def activateBounds(self, varname=None, which_bounds='all'):


1326          """which_bounds argument is either 'all', 'lo', 'hi', or a pair ('lo', 'hi').
 
1327          Calling with no arguments defaults to activating all bounds.""" 
1328          if varname is None and which_bounds=='all': 
1329              self.activateAllBounds = True 
1330          else: 
1331              entry = [0,0] 
1332              if 'hi' in which_bounds: 
1333                  entry[1] = 1 
1334              if 'lo' in which_bounds: 
1335                  entry[0] = 1 
1336              self.activatedBounds[varname] = entry

1337  
 


1338 -    def setInternalVars(self, arg):


1339          if isinstance(arg, list): 
1340              self.forcedIntVars = arg 
1341          elif isinstance(arg, str): 
1342              self.forcedIntVars = [arg]

1343          # !! Should check whether these are valid variable names of model
 
1344  
 


1345 -    def mapEvent(self, hostGen, eventname, target, eventmapping=None):


1346          """eventmapping may be a dictionary or an EvMapping product.
 
1347          You must have declared all generators before calling this function!
 
1348          """ 
1349          allGenNames = [] 
1350          for gname, geninfo in self._generators.iteritems(): 
1351              # geninfo may be an args(dict) type or a GDescriptor
 
1352              if isinstance(geninfo, GDescriptor): 
1353                  allGenNames.append(geninfo.modelspec.name) 
1354              else: 
1355                  allGenNames.append(geninfo['modelspec'].name) 
1356          if target not in allGenNames and target != 'terminate': 
1357              raise ValueError("Unknown target Generator %s"%target) 
1358          if hostGen not in self._generators: 
1359              raise ValueError("Unknown host Generator %s"%hostGen) 
1360          try: 
1361              genEvs = self._events[hostGen] 
1362          except KeyError: 
1363              genEvs = [] 
1364          # hack to allow reference to domain bounds hi and lo events before
 
1365          # their creation
 
1366          is_domev = eventname[-6:] in ['_domlo', '_domhi'] and len(eventname) > 6 
1367          evNames = [ev.name for ev in genEvs] 
1368          if eventname not in evNames and eventname != 'time' and not is_domev: 
1369              raise ValueError("Unknown event '%s' for host Generator"
 
1370                               " '%s'"%(eventname, hostGen)) 
1371          if eventmapping is None: 
1372              evm = EvMapping() 
1373          elif isinstance(eventmapping, dict): 
1374              try: 
1375                  pars = geninfo['modelspec'].pars 
1376              except AttributeError: 
1377                  pars = [] 
1378              evm = EvMapping(eventmapping,
 
1379                              infodict={'vars': geninfo['modelspec'].vars,
 
1380                                        'pars': pars}) 
1381          else: 
1382              evm = eventmapping 
1383          if hostGen in self.eventmaps: 
1384              self.eventmaps[hostGen].append((eventname, (target, evm))) 
1385          else: 
1386              self.eventmaps[hostGen] = [(eventname, (target, evm))]

1387  
 
1388  
 
1389  # ---------------------------------------------------------------------------
 
1390  
 
1391  ## Utility functions
 


1392 -def embed(gen, icdict=None, name=None, tdata=None,
 
1393            make_copy=True):


1394      """Only use this function for building non-hybrid models with single
 
1395      Generators. Otherwise, use the ModelConstructor class.
 
1396  
 
1397      NB The supplied Generator is *copied* into the model unless
 
1398      optional make_copy argument is False.""" 
1399      assert isinstance(gen, Generator.Generator), ("gen argument "
 
1400                                          "must be a Generator object") 
1401      if name is None: 
1402          name = gen.name 
1403      if make_copy: 
1404          g = copy.copy(gen) 
1405      else: 
1406          g = gen 
1407      modelInfoEntry = makeModelInfoEntry(MProject.GeneratorInterface(g),
 
1408                                  [g.name]) 
1409      modelArgs = {'name': name,
 
1410                   'modelInfo': makeModelInfo([modelInfoEntry])} 
1411      modelArgs['ics'] = g.get('initialconditions') 
1412      if icdict is not None: 
1413          # allows for partial specification of ICs here
 
1414          modelArgs['ics'].update(icdict) 
1415      if tdata is not None: 
1416          modelArgs['tdata'] = tdata 
1417      elif g.tdata is not None: 
1418          modelArgs['tdata'] = g.tdata 
1419      return Model.NonHybridModel(modelArgs)

1420  
 
1421  
 


1422 -def makeModelInfo(arg):


1423      if len(arg) == 1 and isinstance(arg, dict): 
1424          dsList = [arg] 
1425      else: 
1426          dsList = arg 
1427      allDSNames = [] 
1428      returnDict = {} 
1429      for infodict in dsList: 
1430          assert len(infodict) == 1, \
 
1431                     "Incorrect length of info dictionary" 
1432          dsName = infodict.keys()[0] 
1433          if dsName not in allDSNames: 
1434              allDSNames.append(dsName) 
1435              returnDict.update(infodict) 
1436          else: 
1437              raise ValueError("clashing DS names in info "
 
1438                               "dictionaries") 
1439          try: 
1440              assert remain(infodict.values()[0].keys(), ['dsi',
 
1441                      'swRules', 'globalConRules', 'domainTests']) == [] 
1442          except AttributeError: 
1443              raise TypeError("Expected dictionary in modelInfo entry") 
1444          except AssertionError: 
1445              raise ValueError("Invalid keys in modelInfo entry") 
1446      return returnDict

1447  
 
1448  
 


1449 -class EvMapping(object):


1450      """Event mapping class, for use by makeModelInfoEntry and, when
 
1451      instantiated, the Model class.
 
1452  
 
1453      assignDict maps its values onto the variable or parameter named by the
 
1454      key. To use the simple syntax in these assignments, either the 'model'
 
1455      argument or the 'infodict' argument must be provided, the first taking
 
1456      preference if both are provided. An instantiated Model object must be
 
1457      provided with the 'model' argument in order to name these variables and
 
1458      parameters without further qualification. A dictionary with keys 'vars'
 
1459      and 'pars' must provide lists of variable and parameter names for the
 
1460      'infodict' argument. Use this argument with ModelConstructor when an
 
1461      instantiated model is not available. With either of these arguments,
 
1462      assignments must be given in the (key, value) form: "a", "1 + a*k/2"
 
1463  
 
1464      Without the model argument, assignments must be given in the (key, value) form:
 
1465       "xdict['a']", "1+xdict['a']*pdict['k']/2"
 
1466  
 
1467      defStrings (list of valid python statements) overrides assignDict if supplied at
 
1468      initialization, to permit full flexibility in the contents of the
 
1469      event mapping function. These strings must use "xdict" and "pdict" to reference
 
1470      the variables and parameters, respectively.
 
1471  
 
1472      Use activeDict to map named events to a given setting for 'active' (Boolean).
 
1473      """ 
1474  
 


1475 -    def __init__(self, assignDict=None, defString="",
 
1476                   activeDict=None, model=None, infodict=None):


1477          if assignDict is None: 
1478              assignDict = {} 
1479          else: 
1480              # parse assignments to use xdict, pdict if model was provided
 
1481              new_assignDict = {} 
1482              if model is None: 
1483                  try: 
1484                      vars = infodict['vars'] 
1485                      pars = infodict['pars'] 
1486                  except: 
1487                      raise ValueError("Must pass dictionary of 'vars' and 'pars'") 
1488              else: 
1489                  try: 
1490                      vars = model.query('vars') 
1491                      pars = model.query('pars') 
1492                  except: 
1493                      raise ValueError("Must pass instantiated Model") 
1494              for key, value in assignDict.items(): 
1495                  rhs = ModelSpec.QuantSpec('rhs', value) 
1496                  rhs_str = '' 
1497                  for tok in rhs.parser.tokenized: 
1498                      if tok in vars: 
1499                          rhs_str += "xdict['%s']"%tok 
1500                      elif tok in pars: 
1501                          rhs_str += "pdict['%s']"%tok 
1502                      else: 
1503                          rhs_str += tok 
1504                  if key in vars: 
1505                      new_assignDict["xdict['%s']"%key] = rhs_str 
1506                  elif key in pars: 
1507                      new_assignDict["pdict['%s']"%key] = rhs_str 
1508                  else: 
1509                      raise ValueError("Invalid LHS for event mapping") 
1510              assignDict = new_assignDict 
1511          if activeDict is None: 
1512              activeDict = {} 
1513          self.assignDict = assignDict.copy() 
1514          self.defString = defString 
1515          self.activeDict = activeDict.copy() 
1516          self.makeCallFn()

1517  
 
1518  
 


1519 -    def __cmp__(self, other):


1520          try: 
1521              return alltrue([self.assignDict==other.assignDict,
 
1522                              self.defString==other.defString,
 
1523                              self.activeDict==other.activeDict]) 
1524          except AttributeError: 
1525              return False

1526  
 


1527 -    def makeCallFn(self):


1528          indent = "  " 
1529          fnString = """def evmapping(self, xdict, pdict, idict, estruct, t):""" 
1530          if self.defString == "" and self.assignDict == {} and self.activeDict == {}: 
1531              # default is the "identity mapping" (do nothing)
 
1532              fnString += indent + "pass\n" 
1533          elif len(self.defString) >= 13 and self.defString[:13] == "def evmapping": 
1534              # already defined, probably rebuilding after save/load object
 
1535              fnString = self.defString 
1536          else: 
1537              if len(self.assignDict) > 0: 
1538                  for lhs, rhs in self.assignDict.iteritems(): 
1539                      if not(type(lhs)==type(rhs)==str): 
1540                          raise TypeError("Assignment dictionary for event "
 
1541                                          "mapping must consist of strings for "
 
1542                                          "both keys and values") 
1543                  fnString += "\n" + indent + ("\n"+indent).join(["%s = %s"%(l,r) \
 
1544                                      for l, r in self.assignDict.items()]) 
1545              if len(self.defString) > 0: 
1546                  fnString += "\n" + indent + ("\n"+indent).join(self.defString.split("\n")) 
1547              if len(self.activeDict) > 0: 
1548                  for evname, state in self.activeDict.iteritems(): 
1549                      if not(type(evname)==str and type(state)==bool): 
1550                          raise TypeError("Invalid types given for setting "
 
1551                                          "active events") 
1552                  fnString += "\n" + indent + \
 
1553                           ("\n"+indent).join(["estruct.setActiveFlag('%s',%s)"%(evname,str(state)) \
 
1554                                      for evname, state in self.activeDict.items()]) 
1555              self.defString = fnString 
1556          try: 
1557              exec fnString 
1558          except: 
1559              print 'Invalid function definition for event mapping:' 
1560              print fnString 
1561              raise 
1562          setattr(self, 'evmapping', types.MethodType(locals()['evmapping'],
 
1563                                                        self, self.__class__))

1564  
 


1565 -    def __getstate__(self):


1566          d = copy.copy(self.__dict__) 
1567          try: 
1568              del d['evmapping'] 
1569          except KeyError: 
1570              print "'evmapping' local function not in self.__dict__" 
1571          return d

1572  
 


1573 -    def __setstate__(self, state):


1574          self.__dict__.update(state) 
1575          self.makeCallFn()

1576  
 
1577  
 


1578 -def makeEvMapping(mappingDict, varnames, parnames):


1579      raise NotImplementedError("Use EvMapping directly now with infodict argument of 'vars' and 'pars' keys")

1580  ##    evMapDict = {}
 
1581  ##    namemap = {}
 
1582  ##    for varname in varnames:
 
1583  ##        namemap[varname] = "xdict['"+varname+"']"
 
1584  ##    for parname in parnames:
 
1585  ##        namemap[parname] = "pdict['"+parname+"']"
 
1586  ##    for k, v in mappingDict.iteritems():
 
1587  ##        v_dummyQ = Symbolic.QuantSpec('dummy', v)
 
1588  ##        v_dummyQ.mapNames(namemap)
 
1589  ##        evMapDict["xdict['%s']"%k] = v_dummyQ()
 
1590  ##    return EvMapping(evMapDict)
 
1591  
 


1592 -def validateTransitionName(name, special_reasons):


1593      if sometrue([name == r for r in special_reasons + ['time', 'terminate']]): 
1594          raise ValueError("Name %s is reserved:\n"%name + \
 
1595              "Cannot use variable names or internal names 'time' and 'terminate'")

1596  
 


1597 -def makeModelInfoEntry(dsi, allModelNames=None, swmap_list=None,
 
1598                         globcon_list=None, nonevent_reasons=None):


1599      """Create an entry for the modelInfo attribute of a Model or Generator,
 
1600      already wrapped in a dsInterface object. Specify the list of non-event based
 
1601      reasons which can be generated by global consistency checks.""" 
1602  
 
1603      if allModelNames is None: 
1604          allModelNames = [] 
1605      if swmap_list is None: 
1606          swmap_list = [] 
1607      if globcon_list is None: 
1608          globcon_list = [] 
1609      if nonevent_reasons is None: 
1610          nonevent_reasons = [] 
1611      assert isinstance(allModelNames, list), \
 
1612                               "'allModelNames' argument must be a list" 
1613      assert isinstance(swmap_list, list), \
 
1614                               "'swmap_list' argument must be a list" 
1615      assert isinstance(globcon_list, list), \
 
1616                               "'globcon_list' argument must be a list of ModelInterfaces" 
1617      assert isinstance(nonevent_reasons, list), \
 
1618                               "'nonevent_reasons' argument must be a list" 
1619  #    assert remain(globcon_list, allModelNames) == [], \
 
1620  #               "global consistency list must consist of declared model names only"
 
1621      doms = {} 
1622      if isinstance(dsi, MProject.GeneratorInterface): 
1623          assert allModelNames == [dsi.model.name], \
 
1624                 "Cannot use non-embedded Generators in hybrid system" 
1625          if swmap_list != []: 
1626              for (name, target) in swmap_list: 
1627                  if isinstance(target, str): 
1628                      if target != 'terminate': 
1629                          print name, target 
1630                          raise AssertionError("Generators can only be used "
 
1631                                               "directly for non-hybrid systems") 
1632                  else: 
1633                      # had better be a pair with first element == name
 
1634                      try: 
1635                          assert target[0] != name 
1636                      except (TypeError, AssertionError): 
1637                          # type error if not subscriptable
 
1638                          print name, target 
1639                          raise AssertionError("Generators can only be used "
 
1640                                               "directly for non-hybrid systems") 
1641          for vname, var in dsi.model.variables.iteritems(): 
1642              if alltrue(var.depdomain.isfinite()): 
1643                  doms[vname] = Model.domain_test(vname+'_domtest',
 
1644                          pars=args(coordname=vname,
 
1645                                    derivname='D_'+vname,
 
1646                              interval=var.depdomain, verbose_level=0)) 
1647          # domain tests here for event-based tests?
 
1648          return {dsi.model.name: {'dsi': dsi, 'domainTests': doms,
 
1649                                 'swRules': {}, 'globalConRules': globcon_list}} 
1650      elif isinstance(dsi, MProject.ModelInterface): 
1651          model = dsi.model 
1652      else: 
1653          raise TypeError("Invalid type for DS interface: "
 
1654                          "must be a GeneratorInterface or ModelInterface") 
1655      # continue here only for ModelInterface
 
1656      for vname, dom in model.query('vardomains').iteritems(): 
1657          if alltrue(dom.isfinite()): 
1658              #vname_compat = model._FScompatibleNames(vname)
 
1659              doms[vname] = Model.domain_test(vname+'_domtest',
 
1660                      pars=args(coordname=vname,
 
1661                                derivname='D_'+vname,
 
1662                          interval=dom, verbose_level=0)) 
1663      # domain tests here for event-based tests?
 
1664      special_reasons = ['time'] + model.query('variables') + nonevent_reasons 
1665      validateTransitionName(model.name, special_reasons) 
1666      try: 
1667          # BUG !!! should only collect terminal events
 
1668          allEndReasonNames = model.query('events').keys() \
 
1669                              + special_reasons 
1670      except AttributeError: 
1671          # no events associated with the model
 
1672          allEndReasonNames = special_reasons 
1673      if model.name not in allModelNames: 
1674          print model.name, allModelNames 
1675          raise ValueError('Sub-model`s name not in list of all '
 
1676                                           'available names!') 
1677      if not alltrue([name not in allEndReasonNames for name in allModelNames]): 
1678          print model.name, allModelNames 
1679          raise ValueError('Sub-model names overlapped with event or '
 
1680                           'variable names') 
1681      allTargNames = allModelNames + ['terminate'] 
1682      # if no event map function specified, assume the identity fn
 
1683      seenReasons = [] 
1684      swmap_pairs = [] 
1685      if swmap_list == []: 
1686          raise ValueError("There must be an event mapping "
 
1687                    "specified when the model is hybrid") 
1688      for mapentry in swmap_list: 
1689          # check the entries of swmap_list and turn into a
 
1690          # (reason, infopair) pair, adding a default event map function
 
1691          # to some entries
 
1692          reason = mapentry[0] 
1693          mapping_info = mapentry[1] 
1694          if len(mapentry) > 2: 
1695              raise ValueError("mapping entry must be (reason, info-pair) tuple") 
1696          if isinstance(mapping_info, tuple): 
1697              targetName = mapping_info[0] 
1698              numargs = len(mapping_info) 
1699          elif isinstance(mapping_info, str): 
1700              targetName = mapentry[1] 
1701              numargs = 1 
1702          else: 
1703              raise TypeError("Invalid event mapping entry") 
1704          if numargs == 2: 
1705              epmap = mapping_info[1] 
1706              assert isinstance(epmap, EvMapping), "Must supply EvMapping class" 
1707              swmap_pairs.append((reason, mapping_info)) 
1708          elif numargs == 1: 
1709              # use default identity mapping fn for event
 
1710              # and make this entry into a three-tuple
 
1711              swmap_pairs.append((reason, (targetName, EvMapping()))) 
1712          else: 
1713              raise ValueError("Expected 2 or 3 arguments to model "
 
1714                               "switch map entry") 
1715          assert reason not in seenReasons, ('reason cannot appear more than'
 
1716                                             ' once in map domain') 
1717          seenReasons.append(reason) 
1718          if reason not in allEndReasonNames: 
1719              print "Model %s:"%model.name 
1720              print allEndReasonNames 
1721              raise ValueError("name '"+reason+"' in map "
 
1722                                              "domain is missing") 
1723          if targetName not in allTargNames: 
1724              print "Model %s:"%model.name 
1725              print allTargNames 
1726              raise ValueError("name '"+targetName+"' in "
 
1727                                              "map range is missing") 
1728      unseen_sr = remain(allEndReasonNames, seenReasons) 
1729      if unseen_sr != []: 
1730          # then there are 'end reasons' that do not have switch rules,
 
1731          # so give them defaults (terminate) - must use empty EvMapping
 
1732          # to match how the others will be created internally
 
1733          for r in unseen_sr: 
1734              swmap_pairs.append((r, ('terminate', EvMapping()))) 
1735      if len(swmap_pairs) != len(allEndReasonNames): 
1736          info(dict(swmap_pairs)) 
1737          print "(%i in total), versus:"%len(swmap_pairs) 
1738          print allEndReasonNames, "(%i in total)"%len(allEndReasonNames) 
1739          sw_keys = dict(swmap_pairs).keys() 
1740          print remain(sw_keys, allEndReasonNames) 
1741          print remain(allEndReasonNames, sw_keys) 
1742          raise ValueError('Incorrect number of map pairs given in argument') 
1743      return {model.name: {'dsi': dsi, 'domainTests': doms,
 
1744                  'swRules': dict(swmap_pairs), 'globalConRules': globcon_list}}

1745  
 
1746  
 


1747 -def processReused(sourcenames, auxvarnames, flatspec, registry,
 
1748                    FScompatibleNames, FScompatibleNamesInv):


1749      """Find and process reused terms in abstract specification. To avoid
 
1750      RHS specs depending on auxiliary variables, temp variables will be declared
 
1751      in FuncSpec.py and used in both the RHS and auxiliary variables in the
 
1752      target language specification.
 
1753      """ 
1754      reuseTerms={} 
1755      subsExpr={} 
1756      num_reused=0 
1757      # auxvarnames are those that sourcename definitions cannot use
 
1758      # build auxiliary token map to get rid of auxvar - auxvar inter-
 
1759      # dependencies
 
1760      u_subsMap = {} 
1761      for auxtok in auxvarnames: 
1762          tokobj = registry[FScompatibleNamesInv(auxtok)].obj 
1763          addtokbraces = tokobj.spec.isCompound() 
1764  #        u_new_reusedname = "__"+auxtok+str(num_reused)
 
1765          FScompat_spec = "".join(FScompatibleNames(tokobj.spec[:])) 
1766          u_subsMap[auxtok] = "("*addtokbraces + \
 
1767                         FScompat_spec + ")"*addtokbraces 
1768  #        u_subsMap[auxtok] = "".join(FScompatibleNames( \
 
1769  #                                     tokobj.spec[:]))
 
1770      # some of these u_subsMap targets may contain auxiliary variables
 
1771      # themselves, so we must purge them now in repeated passes to u_subsMap.
 
1772      # put in a trap for infinite loop of inter-dependencies!
 
1773      loopCount = 0 
1774      loopMaxDepth = 15 
1775      purgeDone = {}.fromkeys(auxvarnames, False) 
1776      while not all(purgeDone.values()) and loopCount < loopMaxDepth: 
1777          loopCount += 1 
1778  #        print "Loop count: ", loopCount
 
1779          tempMap = {} 
1780          for auxtok, sx in u_subsMap.iteritems(): 
1781  #            print "** ", auxtok
 
1782              if purgeDone[auxtok]: 
1783  #                print "  Continue 1"
 
1784                  continue 
1785              dummyQ = Symbolic.QuantSpec('dummy', sx) 
1786              if not any([auxname in dummyQ \
 
1787                              for auxname in auxvarnames]): 
1788                  # no auxvar names appear in the subs expr, so this is cleared
 
1789                  purgeDone[auxtok] = True 
1790  #                print "  Continue 2"
 
1791                  continue 
1792              dummyQ.mapNames(u_subsMap) 
1793              tempMap[auxtok] = dummyQ() 
1794          # update name map with any new substitutions
 
1795  #        if tempMap != {}:
 
1796  #            info(tempMap)
 
1797          u_subsMap.update(tempMap) 
1798      if not purgeDone and len(auxvarnames)>0: 
1799          # then must have maxed out
 
1800          print "Declared auxilary variables:", auxvarnames 
1801          raise RuntimeError("You probably have an infinite loop of auxiliary "
 
1802                         "variable inter-dependencies: recursion depth of "
 
1803                         "more than %i encountered during model build"%loopCount) 
1804      for v in sourcenames: 
1805          if v not in flatspec['vars']: 
1806              # v could be a parameter, a function name, or a constant (in a
 
1807              # recursive call), so ignore
 
1808              continue 
1809          subsMap = {} 
1810          dummyQ = Symbolic.QuantSpec('dummy', flatspec['vars'][v]) 
1811          for u in dummyQ.usedSymbols: 
1812              if u in auxvarnames: 
1813                  new_reusedname = "__"+u 
1814                  if new_reusedname in reuseTerms.values(): 
1815                      # simple way to avoid name clashes
 
1816                      new_reusedname += '_'+str(num_reused) 
1817                      num_reused += 1 
1818                  spec_text = flatspec['vars'][u] 
1819                  testQ = Symbolic.QuantSpec('dummy', spec_text) 
1820                  testQ.mapNames(mathNameMap) 
1821                  # add test for unary minus otherwise no braces around
 
1822                  # testQ will lead to both signs disappearing on reuseTerm
 
1823                  # substitution, leaving two symbols adjoined without any
 
1824                  # operator!
 
1825                  addbraces = testQ.isCompound() or testQ()[0] == '-' 
1826                  # no subs expression for auxvar that points to a constant
 
1827                  noSubsExpr = not addbraces and \
 
1828                        (FScompatibleNamesInv(spec_text) in registry \
 
1829                                            or isNumericToken(spec_text)) 
1830                  # make substitutions for any aux vars appearing in
 
1831                  # spec_text (testQ)
 
1832                  testQ.mapNames(u_subsMap) 
1833                  # update addbraces after mapping
 
1834                  addbraces = testQ.isCompound() or testQ()[0] == '-' 
1835                  #testQ.simplify()
 
1836                  spec_text_new = "("*addbraces + testQ() + ")"*addbraces 
1837  #                spec_text_new = testQ()
 
1838                  if not noSubsExpr: 
1839                      if u in subsExpr: 
1840                          # putting braces around auxtok in u_subsMap means
 
1841                          # that some of the expressions won't have the same
 
1842                          # bracketing as spec_text_new, so don't bother with
 
1843                          # this check
 
1844                          pass 
1845  #                        if subsExpr[u] != spec_text_new:
 
1846  #                            print subsExpr[u]
 
1847  #                            print spec_text_new
 
1848  #                            raise RuntimeError("Different subs expr for %s in subsExpr"%u)
 
1849                      else: 
1850                          subsExpr[u] = spec_text_new 
1851                      if testQ()[0] == '-': 
1852                          reuse_term = spec_text_new 
1853                      else: 
1854                          reuse_term = testQ() 
1855                      if reuse_term not in reuseTerms: 
1856                          reuseTerms[reuse_term] = new_reusedname 
1857                  if u in subsMap: 
1858                      raise RuntimeError("%s already in subsMap!"%u) 
1859                  else: 
1860                      subsMap[u] = spec_text_new 
1861          # use QuantSpec's inbuilt tokenized version of exp_var definition
 
1862          # to make substitutions using the name mapping subsMap
 
1863          dummyQ.mapNames(subsMap) 
1864          #dummyQ.simplify()
 
1865          # uses addvbraces is use addbraces above, otherwise get clash
 
1866  ##        addvbraces = dummyQ.isCompound()
 
1867  ##        subsExpr[v] = "("*addvbraces + dummyQ() + ")"*addvbraces
 
1868          dummyQ.mapNames(mathNameMap) 
1869          subsExpr[v] = dummyQ() 
1870      return reuseTerms, subsExpr

1871
```

  


| Home | Trees | Indices | Help | PyDSTool |
| --- | --- | --- | --- | --- |

|  |  |
| --- | --- |
| Generated by Epydoc 3.0.1 on Fri May 4 15:24:22 2012 | http://epydoc.sourceforge.net |
